# The Critical Role of Comorbidities in Managing Heart Failure with Preserved Ejection Fraction (HFpEF)

**DOI:** 10.1002/ehf2.15169

**Published:** 2024-11-16

**Authors:** Piotr Gajewski, Robert Zymlinski, Jan Biegus

**Affiliations:** ^1^ Institute of Heart Diseases Wroclaw Medical University Wrocław Poland

Heart failure with preserved ejection fraction (HFpEF) represents an increasingly prevalent and challenging phenotype of heart failure (HF), primarily due to the multitude of comorbidities that both contribute to its pathogenesis and complicate its management. As the population ages and diagnostic measures advance, the rate of HFpEF diagnosis is on the rise, revealing a complex clinical picture where HF often coexists with other chronic conditions. It is essential to distinguish between complications directly related to HF and independent diseases that co‐occur in the same patients, as this distinction is fundamental to tailoring individualized therapeutic strategies.^1^, ^2^, ^3^, ^4^, ^5^, ^6^

Comorbidities in HFpEF not only exacerbate the disease's clinical severity but also accelerate the decline in functional status according to the NYHA classification, amplifying adverse treatment outcomes, including increased mortality and hospitalization rates.^2^, ^3^, ^7^ This editorial highlights the intricate interplay between HFpEF and its common comorbidities, emphasizing the need for a holistic approach to patient management in this context.

One key challenge in managing HFpEF is differentiating it from conditions that mimic its clinical presentation, such as infiltrative cardiomyopathies, coronary artery disease, lung disease, and non‐cardiac conditions like anxiety, depression, severe obesity, and physical deconditioning. Misdiagnosis or delayed diagnosis can lead to ineffective treatments and unnecessary interventions. Following structured diagnostic algorithms is essential to accurately identify HFpEF and optimize treatment pathways. To address these complexities, we have developed a dedicated virtual issue in *ESC Heart Failure*, compiling significant research on HFpEF and encouraging contributions that advance understanding and improve management strategies for these patients.

One of the most widely recognized comorbidities associated with HFpEF is atrial fibrillation (AF), which affects roughly two‐thirds of HFpEF patients. This association is not coincidental but is based on shared pathophysiological mechanisms and risk factors.^8^ AF in HFpEF patients worsens exertional limitations and symptom burden compared to those in sinus rhythm, and it is associated with increased mortality and higher rates of cardiovascular hospitalization and rehospitalization.^9^ Importantly, while AF may not directly increase the risk of sudden death, it accelerates HFpEF progression, impacting each stage of the disease. Furthermore, there are sex‐specific survival differences among patients with both HFpEF and AF, with higher mortality observed in men despite a higher prevalence of the condition in this group. Thus, effective management of AF in HFpEF is crucial, although no standardized therapeutic guidelines currently exist. Catheter ablation shows promise as a potential intervention for symptom alleviation and quality of life improvement, yet its efficacy in HFpEF appears lower than in HF with reduced ejection fraction (HFrEF). Further research is needed to refine intervention strategies for this unique HF phenotype.^10^, ^11^, ^12^

In recent decades, the impact of adipose tissue on cardiovascular health has been extensively studied, highlighting its significant role in HF pathophysiology. In HFpEF, obesity contributes to cardiovascular dysfunction through mechanisms such as neurohormonal activation, hemodynamic overload, oxidative stress, and low‐grade chronic inflammation. Epidemiological studies reveal that HFpEF is more commonly associated with obesity than HFrEF, particularly in Europe and North America, where obesity rates are highest. Interestingly, different patient subgroups exhibit distinct characteristics; younger (<64 years) HFpEF patients with obesity are typically male and present with poorer glycemic control, whereas older (>65 years) HFpEF patients with obesity are predominantly female and often have more severe comorbidities. This underscores the critical role of obesity not only as a risk factor for HF but as a driver of the HFpEF phenotype specifically.^5^, ^13^, ^14^

The interaction between HFpEF and obesity is further complicated by the presence of additional comorbidities like chronic kidney disease (CKD), chronic obstructive pulmonary disease (COPD), various forms of anemia, and obstructive sleep apnea (OSA), each of which independently worsens prognosis. The recognition of obesity as a modifiable risk factor has prompted interest in pharmacological interventions, particularly GLP‐1 agonists, which have shown promise in managing HFpEF‐related obesity. These medications have been associated with reductions in C‐reactive protein (CRP) levels, declines in NT‐proBNP concentrations, weight loss, and decreases in the need for loop diuretics to control HF symptoms. Patients on GLP‐1 agonists report improvements in exercise tolerance and symptom severity, further validating their potential role in HFpEF management.^15^, ^16^

Another crucial area of concern is the assessment of anemia, a common comorbidity in HFpEF associated with poor clinical outcomes. Recent studies suggest that patients who recover from an anemic state or maintain stable hemoglobin levels within the first‐year post‐discharge experience better outcomes compared to those whose anemia worsens. Factors such as female sex, COPD, and better renal function are associated with improved anemia status, whereas advanced age, low body mass index, frailty, and lower initial hemoglobin levels predict anemia deterioration. These findings highlight the need for targeted monitoring and management of hemoglobin levels in elderly HFpEF patients, particularly as anemia is highly prevalent in this group. Adjusting hemoglobin targets below conventional WHO thresholds could lead to more precise treatment in this population, acknowledging the unique challenges anemia presents in HFpEF management.^17^

Recent findings from a cross‐sectional study reveal a significant association between kidney dysfunction—even at mild levels—and diastolic dysfunction, as well as heart failure with preserved ejection fraction (HFpEF). This association was observed independently of other cardiovascular risk factors, suggesting that early kidney impairment could contribute to elevated cardiac filling pressures, a precursor to symptomatic heart failure. The study also highlights that females exhibit a higher prevalence of HFpEF, though no sex‐specific differences were found in the association between kidney dysfunction and diastolic function. Notably, mild renal dysfunction correlated with higher E/e’ ratios, a key indicator of elevated filling pressures, even after excluding participants with existing heart failure. The findings point to a critical need for early intervention in high‐risk individuals, focusing on modifiable factors such as pressure overload, volume management, and systemic inflammation, to potentially prevent progression to HFpEF. The study further underscores the value of utilizing new, race‐neutral equations for estimating glomerular filtration rates (eGFR), which enhances precision across diverse populations. Given that most prior studies focused on populations with advanced chronic kidney disease, this research fills a crucial gap, underscoring the importance of comprehensive cardiac and renal assessments in patients with early or mild renal impairment. Future investigations are warranted to explore whether therapies like RAAS inhibitors, SGLT2 inhibitors, or anti‐inflammatory agents could offer protective benefits against heart failure progression in patients with mild kidney dysfunction.^18^

Lastly, the intersection of HF and stroke represents a high‐risk condition demanding close clinical attention. HF patients have an elevated risk of stroke, particularly in the first 30 days post‐HF diagnosis and during episodes of acute decompensated heart failure (ADHF). This heightened risk is due to various HF‐related factors, including age, disease severity, and the presence of AF. Moreover, stroke risk extends beyond overt events to include silent brain lesions. Conversely, HF is common among patients who suffer from ischemic strokes, either as a pre‐existing condition or as acute hemodynamic decompensation triggered by the stroke. Stroke in HF patients is frequently caused by cardioembolism, though hypoperfusion‐related mechanisms are also relevant. The procoagulant state in HF, encompassing slow blood flow, endothelial activation, and coagulation, underscores the need for a multidisciplinary approach to manage these high‐risk patients effectively.^19^, ^20^

In summary, comorbidities in HFpEF are not mere secondary issues but play a pivotal role in disease progression and patient outcomes. Given the absence of specific, effective therapies for HFpEF, managing comorbid conditions offers a viable strategy to improve patient quality of life and potentially enhance clinical outcomes. Current HF guidelines emphasize the importance of recognizing non‐cardiovascular comorbidities in HFpEF, urging a comprehensive patient management approach that extends beyond cardiac function alone. We hope this special issue of *ESC Heart Failure* inspires continued research on HFpEF and invites contributions that delve into the intricate connections between HFpEF and its comorbidities, ultimately aiming to optimize care and outcomes for this growing patient population.

## Conflict of interest

All authors declare no conflict of interest.
